# Research prospect of human salivary cortisol: a bibliometric analysis

**DOI:** 10.3389/fpsyg.2025.1552821

**Published:** 2025-03-17

**Authors:** Shuo Qin, Juan Liu, Zhe Qin, Jing Jia

**Affiliations:** ^1^Third Clinical School of Medicine, Jinzhou Medical University, Jinzhou, China; ^2^Department of Stomatology, Shandong Health Group, Zaozhuang Hospital, Zaozhuang, China; ^3^Department of Stomatology, Third Medical Center, Chinese PLA General Hospital, Beijing, China

**Keywords:** salivary cortisol, a bibliometric analysis, co-occurrence network analysis, cooperative network analysis, clustering analysis

## Abstract

**Background:**

Salivary cortisol has garnered increasing attention; the aim of this study was to employ bibliometric analysis to identify key papers in this research field and to explore its current status and trends.

**Methods:**

Data were sourced from Clarivate Analytics’ Web of Science core database, utilizing the search terms ‘TS = (‘Salivary cortisol’) AND (Human) NOT (animal)’. All articles published from January 1, 2004, to September 30, 2024, were included.

**Results:**

A total of 876 articles were identified. The United States has made a significant contribution to this field, with the highest number of publications at 291. The University of California system leads this research area, having published 40 articles. Professor Clemens Kirschbaum has authored 21 articles in this domain. The journal Psychoneuroendocrinology published the largest number of articles, totaling 99, which accounts for 11.3% of the overall articles. Additionally, Psychoneur-oendocrinology was the most cited journal, with 3,681 citations. High-frequency keywords reveal the developmental status and future trends of human salivary cortisol. As a biomarker and signaling molecule, salivary cortisol is closely linked to human gender, personality, psychology, and physiology. We identified that “mental health,” “circadian rhythm,” and “assay” may become focal points of interest in the coming years.

**Conclusion:**

Global publications related to human salivary cortisol were systematically reviewed. CiteSpace and VOSviewer were utilized to analyze their bibliometric characteristics, identify the most cited articles in the field, and determine the leading countries, authors, and institutions, along with the interconnections among them. This analysis aims to elucidate the current status, hotspots, and trends in global research, while providing future development directions for scientific inquiry and medical practitioners.

## 1 Introduction

Cortisol, a crucial glucocorticoid hormone in the human body, is present in blood, urine, saliva, and hair, and serves as the final product of the hypothalamic–pituitary–adrenal (HPA) axis. Often referred to as the ‘stress hormone’, cortisol exhibits a strong correlation with stress (Juster et al., 2010). The proportion of salivary cortisol to total cortisol ranges from approximately 1–2% at the lower boundary to 8–9% at the upper boundary (Hellhammer et al., 2009). Salivary cortisol has been demonstrated to be a reliable and convenient biomarker, widely utilized in the study of clinical and psychological disorders (Kirschbaum and Hellhammer, 1994). This reliability is attributed to its positive correlation with plasma cortisol concentration and the non-invasive nature of its collection (Kudielka et al., 2009), which minimizes the risk of artificially elevating cortisol levels due to the stress associated with blood collection (Blair et al., 2017). Consequently, salivary cortisol has emerged as a valuable bioindicator for disease detection.

As a crucial glucocorticoid hormone in the body, it exerts a wide range of effects on mood, health, and the maintenance of connective tissues, making it a common biosignaling molecule in studies assessing psychological stress. In psychosomatic disorders such as anxiety (Grace et al., 2022), depression (Fiksdal et al., 2019), traumatic stress disorder (Pan et al., 2018), dementia (Cho et al., 2024), bi-directional affective disorder (Mukherjee et al., 2022), and others, the levels of salivary cortisol in patients differ from those in healthy individuals, establishing it as an important biomarker for detecting psychosomatic disorders. Studies have shown that following experiences of life stress, cortisol levels increase while cognitive function in patients declines. There exists a significant relationship between cortisol and cognitive function in patients after they encounter stressful life events (Giannouli, 2024). In the context of endocrine system disorders, most of the cortisol found in saliva is in its free form, which possesses considerable biological activity. Salivary cortisol is more suitable for assessing adrenocortical function compared to blood cortisol (Hellhammer et al., 2009). In recent years, cortisol has been regarded as a superior first-line indicator for evaluating the sensitivity and specificity of adrenocortical function (Tell et al., 2014). Additionally, late-night salivary cortisol has demonstrated superior diagnostic performance as a primary biochemical test for Cushing’s syndrome (Elias et al., 2014). Salivary cortisol levels, influenced by chronic stress and endocrine stress in diabetic patients, significantly inversely correlate with poor glucose tolerance and insulin resistance (Siddiqui et al., 2015). In the context of cardiovascular disease, salivary cortisol serves as a predictor for the risk of coronary heart disease (Degroote et al., 2023), demonstrates potential clinical utility in assessing pulmonary hypertension (Tulloh et al., 2020), and is found to be elevated in hypertensive patients compared to those with normal blood pressure (Yan et al., 2019). Furthermore, salivary cortisol levels are closely linked to various oral diseases, including periodontal disease (Lee et al., 2023), lichen planus (Pippi et al., 2014), maxillofacial pain (Jasim et al., 2014), burnt mouth syndrome (Nosratzehi et al., 2017), bruxism (Fritzen et al., 2022), and temporomandibular joint disorders (Da Silva et al., 2008). As a biosignaling molecule, the measurement of salivary cortisol concentrations holds significant importance for the diagnosis and treatment of these conditions.

Bibliometric analysis is a technique that employs mathematical and statistical methods to quantitatively examine the size, structure, distribution, citation relationships, and other characteristics of academic literature. Its primary objective is to elucidate the development trends, research hotspots, knowledge networks, modes of research cooperation, and academic influence within a specific discipline by analyzing literary data. This analysis aims to provide data support for scientific research management, discipline planning, and policy-making. A traditional literature review typically involves a qualitative analysis that provides a subjective summary, relying on inductive reasoning to synthesize a selective range of literature. This process is dependent on the researchers’ subjective evaluations to derive summary views, critical assessments, and theoretical integrations. In contrast, bibliometrics employs quantitative data analysis of extensive literature, utilizing statistical indicators. The results of bibliometric analysis can be reused to uncover macro trends, rules, and network relationships. Based on bibliometrics (Groos and Pritchard, 1969), a quantitative analysis of the research progress concerning human salivary cortisol can yield detailed information about the authors of salivary cortisol articles (Mayr and Scharnhorst, 2014), as well as associated keywords, journals, countries, institutions, references, and other relevant details. Co-citation is frequently employed in bibliometric analyses; it is defined as a relationship visualization analysis when two articles are cited by one or more other articles simultaneously (Ma and Xi, 1992). This underscores the importance of the visualization method in co-citation analysis, which aids in data interpretation and leads to more comprehensive results. The aim of this bibliometric study was to systematically assess the progress of research on human salivary cortisol. Through this analysis, we seek to elucidate the trajectory of research development, highlight significant findings, and emphasize the role of salivary cortisol as both a biomarker and a signaling molecule in disease progression. Ultimately, this work aspires to inform future research directions and support the integration of salivary cortisol into clinical practice, thereby enhancing early detection, personalized treatment, and monitoring of various diseases.

## 2 Methods

### 2.1 Sources and searches

**Figure 1:**
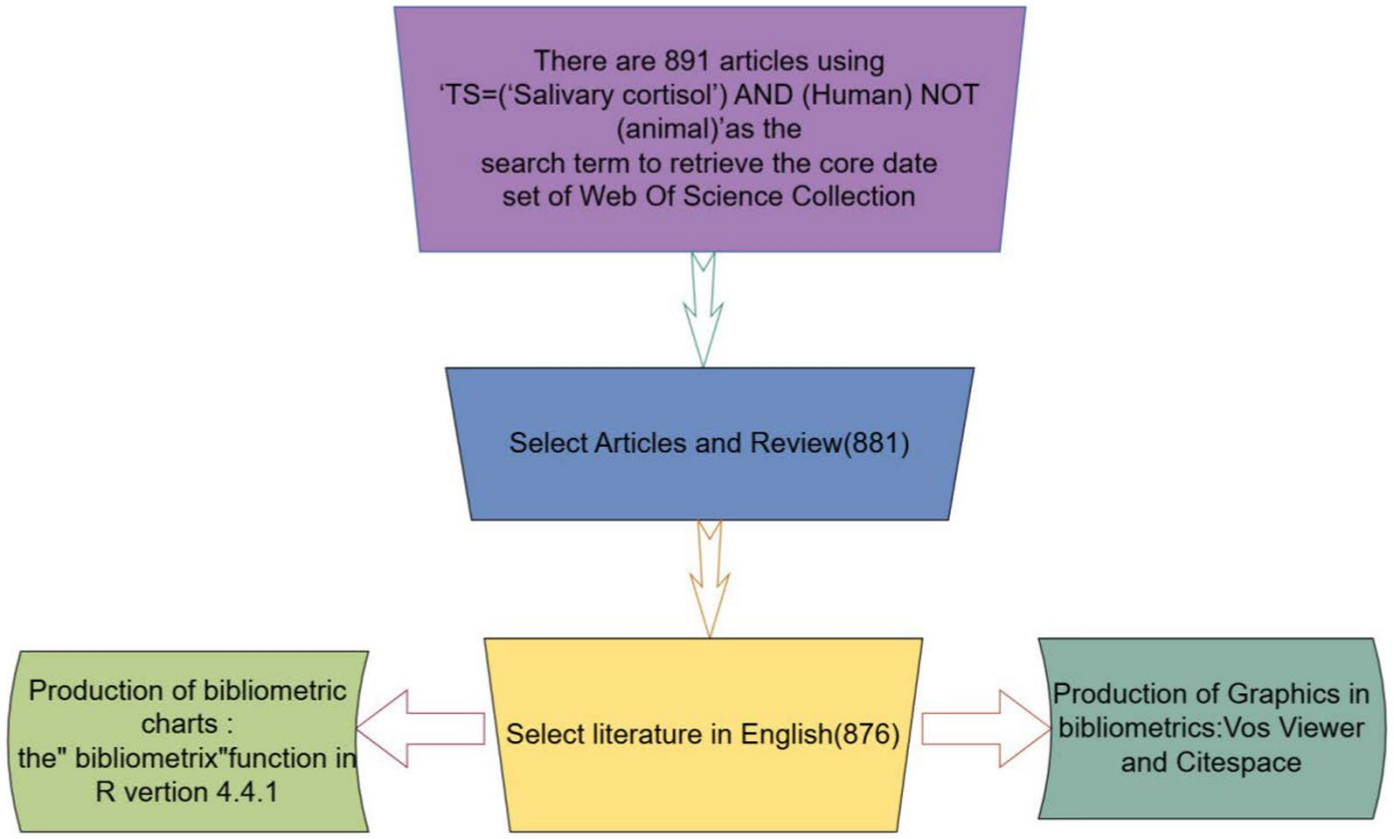
Flow chart.

We used data from Clarivate Analytics’ Web of Science core database for our study. This high-quality database of digital literature resources has been accepted by a wide range of researchers as the most suitable database for bibliometric analysis. This high-quality digital literature resource has been accepted by a wide range of researchers as the most suitable database for bibliometric analysis. we used ‘TS = (‘Salivary cortisol’) AND (Human) NOT (animal)’ as the search term to find all the literature from 1st January 2004 to 30th September 2024. Literature. Articles and Review Articles were selected, written in English, and the flowchart is shown in Figure 1. This study was conducted in accordance with the guidelines of the Declaration of Helsinki.

### 2.2 Data analysis and visualization

Two independent researchers conducted this study to ensure the reliability of the results. The retrieved literature was downloaded in ‘plain text’ format and the corresponding information was extracted for data analysis. Author, country or region, institution, keywords and reference data were analysed and mapped using VOSviewer 1.6.20 and CiteSpace 6.3.R2: Literature Analysis software. Data were imported and analysed in R4.2.2 (R Foundation for Statistical Computing, Vienna, Austria, http://www.R-project.org/) between collaborating countries and surrounding research hotspots. Use WPS Office to analyse the number of articles published by country or region and graphically display trends in the number of articles published. Scimago Graphica, Pajek and other software assisted in image design and data conversion. Figdraw software was used to draw the flow chart.

## 3 Results

### 3.1 Annual publications and trends

**Figure 2:**
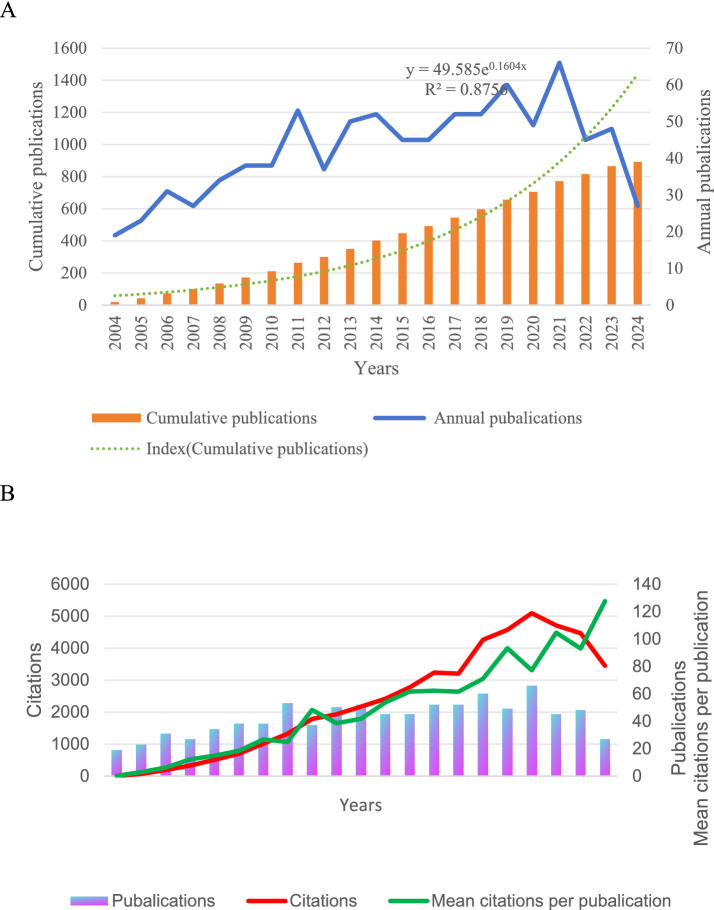
**(A)** The annual number of publications and the cumulative number of publications. **(B)** Annual publications, citations, and average citations.

We included 876 studies, 107 reviews, and 745 articles covering 60 countries and 2,883 organizations. In Figure 2A, we found that the overall number of literature publications from 2004 to 2021 has been increasing, and the cumulative number of articles has been increasing and showing an exponential curve (y = 49.585e^01604x^, R2 = 0.8756), indicating that with the continuous progress of economy and science and technology, there is more and more in-depth research on human salivary cortisol. Although the number of annual publications in 2007, 2012, 2015, 2016, 2020, and 2022 fell back a bit, the overall increase in the number of articles compared with the pre-2005 period may be related to the interests of various scholars, competitive relationships, and technological turnover. From 2004 to 2017, there was an increase in research on human salivary cortisol, with more citations in the literature, and research scholars showed strong interest. However, the number of citations from 2017 to 2018, as shown in Figure 2B, is relatively flat, which may be related to the continuous exploration of new technologies and discoveries among scholars and institutions. From 2018 to 2021, there was a peak period, and the research on cortisol greatly attracted various countries and scholars to collaborate with each other. 2021 to present, the number of citations relatively declined, which may be related to the outbreak of The COVID-19 pandemic and the decrease in the cooperation between institutions in various countries. The average number of citations per article shows an increasing trend over time.

### 3.2 National/regional, institutional issuance

**Figure 3:**
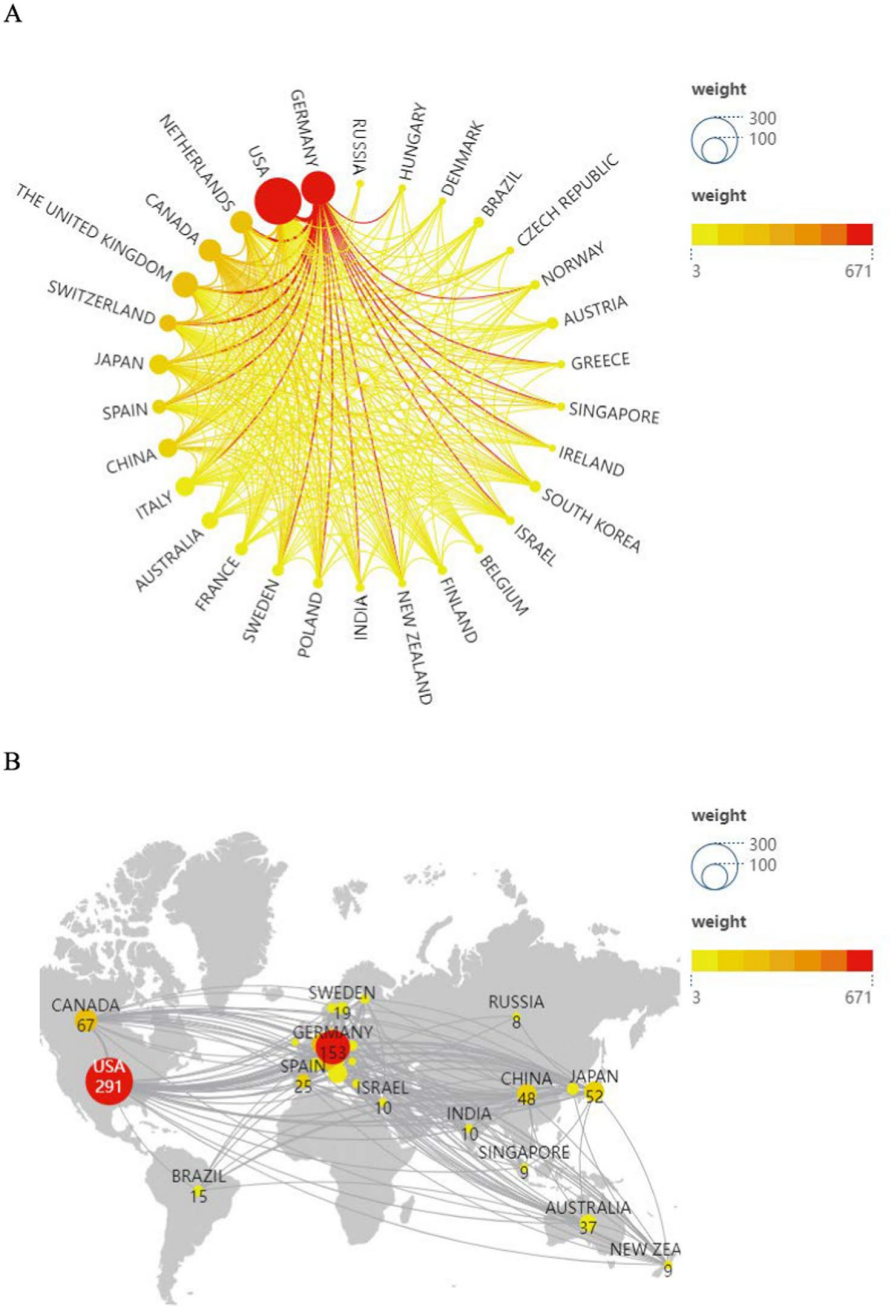
**(A)** Country cooperation chart. **(B)** Visual map.

A total of 60 countries or regions published such studies, and the top 10 countries in terms of the number of articles published are shown in Figure 3B. The main concentration was in North America and Western Europe. The United States published the largest number of 291 articles, followed by Germany (153), the United Kingdom (87), Canada (67), the Netherlands (66), Japan (52), Italy (49), China (48), Australia (57), Switzerland (39), and Australia (37). This is mainly due to the earlier research among these countries, the availability of advanced scientific instruments, and the deep cooperation among the countries. Figure 3A clearly shows the change in the number of publications between countries and the change in the intensity of cooperation between countries, with the United States having the most frequent cooperative relationship with each country, and Germany having the highest total intensity of cooperation between other countries. Figure 3B shows not only the amount of articles sent by each country, but also the close cooperation between countries, a phenomenon reflected in the United States, European countries, China, Australia, Brazil, South Korea, Japan, and New Zealand in particular.

**Figure 4:**
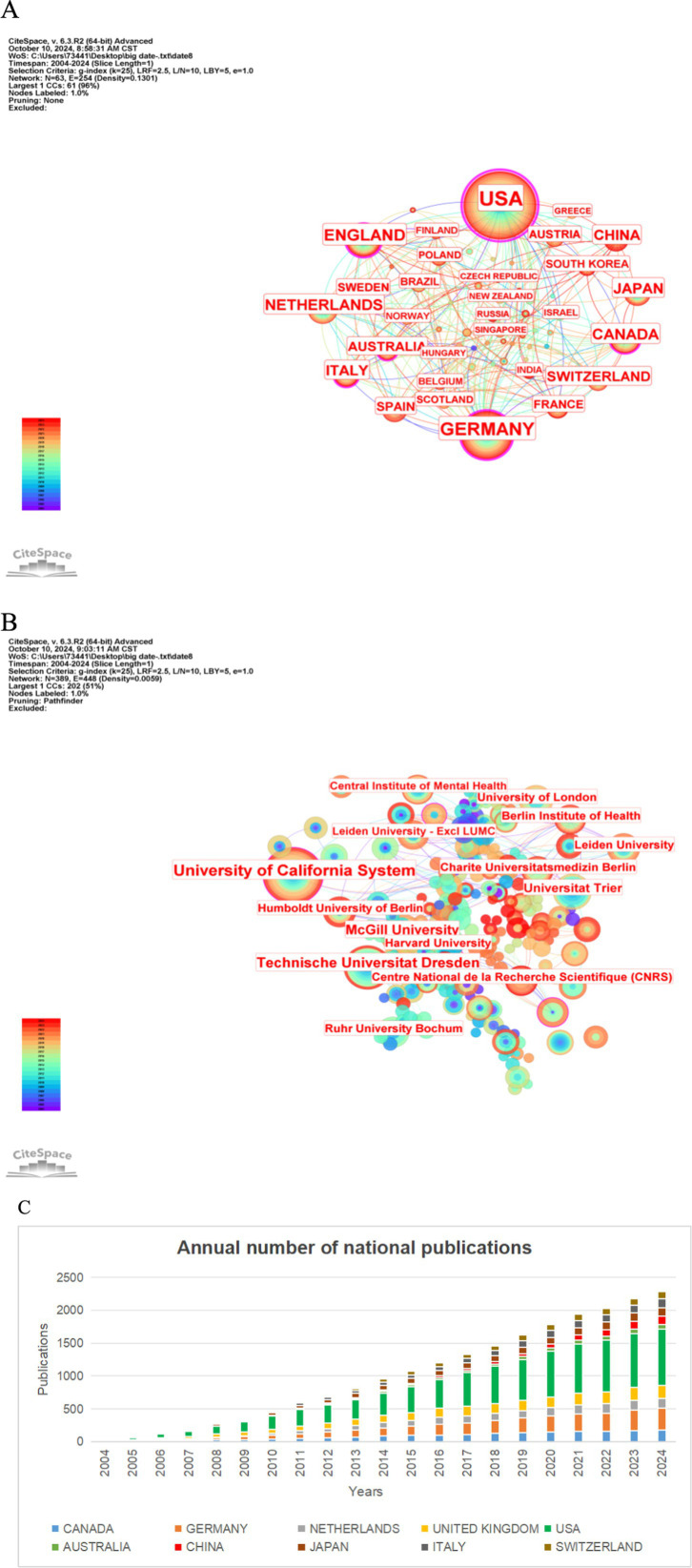
**(A)** States. **(B)** Institutions. **(C)** Annual national publications.

Partnerships across all countries are shown in Figures 3B, 4A. The size of the circle indicates the number of published texts, the thickness of the line indicates the closeness of the connection between the two parties, and centrality is a measure of the importance of a node in the path connecting any pair of nodes in the network. CiteSpace uses a purple ring to indicate the nodes with high centrality, and the thickness of the ring indicates the strength of the centrality, in which the United States is at the center of all the countries’ partnerships, followed by Germany, which is closely related to the strong economic and technological strength of the United States is inseparable.

Of the 2,883 institutions that issue certificates, the top 10 institutions come from seven countries These 10 institutions are the University of California System (40), Technische Universitat Dresden (27), McGill University (20), Universitat Trier (15), University of London (14), Ruhr University Bochum (12), Leiden University (12), Humboldt University of Berlin (11), Center National de la Recherche Scientifique (CNRS) (11), University of Zurich (10). The United States is located in the first place in the world in both the total number of publications and the number of publications per year as shown in Figures 3B, 4C, and we also find that the institutions with the highest number of publications match the top five countries in terms of the number of publications, with one institution in the United States, four institutions in Germany, and one in each of the United Kingdom, Canada, and the Netherlands, although the institutions are more dispersed. The links between institutions in all countries are shown in Figure 4B. The largest issuing institution is the University of California System (40), and several U.S. institutions are closely linked to all of them. The number and proportion of the top ten issuing countries and institutions are listed in Table 1.

**Table 1:** The top 10 countries and institutions.

Rank	Country	Documents (%)	Affiliations	Count (%)
1	USA	291 (24.2%)	University of California System	40 (4.6%)
2	Germany	153 (12.7%)	Technische Universitat Dresden	27 (3.1%)
3	The United Kingdom	87 (7.2%)	McGill University	20 (2.3%)
4	Canada	67 (5.6%)	Universitat Trier	15 (1.7%)
5	Netherlands	66 (5.4%)	University of London	14 (1.6%)
6	Japan	52 (4.3%)	Ruhr University Bochum	12 (1.4%)
7	Italy	49 (4.1%)	Leiden University	12 (1.4%)
8	China	48 (4.0%)	Humboldt University of Berlin	11 (1.3%)
9	Switzerland	39 (3.2%)	Centre National de la Recherche Scientifique (CNRS)	11 (1.3%)
10	Australia	37 (3.1%)	University of Zurich	10 (1.1%)

### 3.3 Journal and co-cited journal analysis

**Figure 5:**
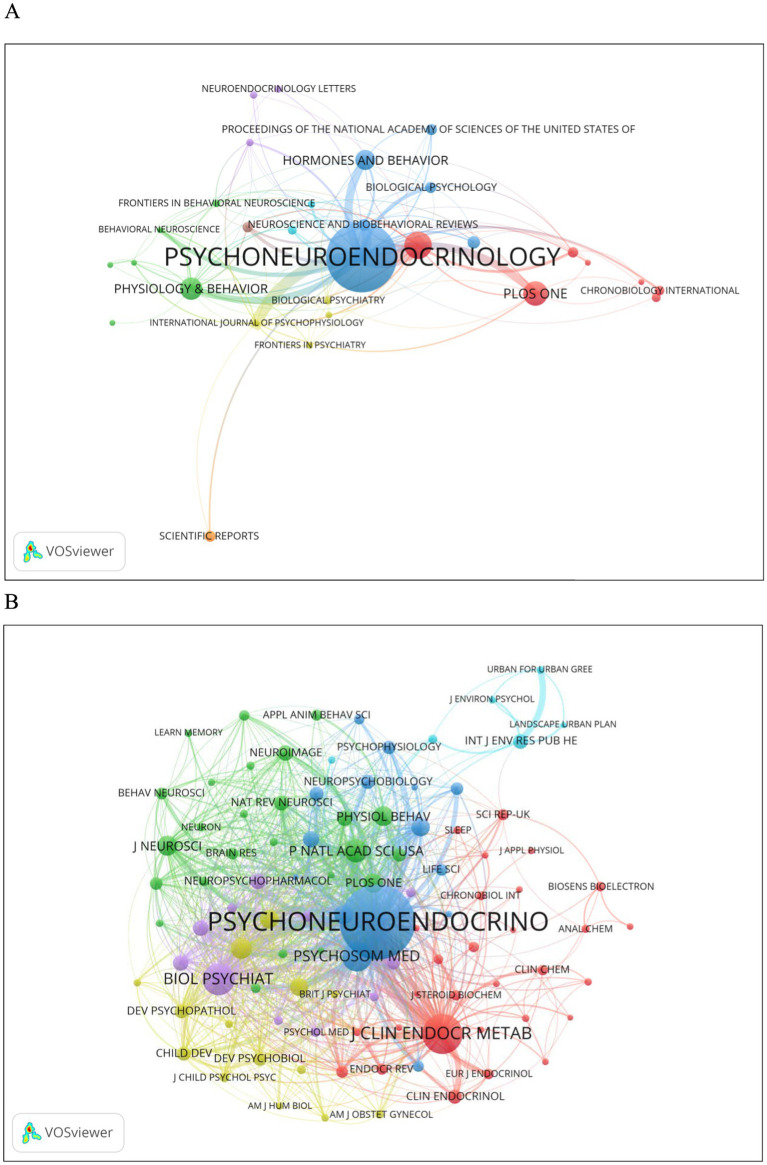
**(A)** Journal. **(B)** Co-cited journal analysis.

Articles published up to 09/2024 were published in 372 journals. The largest number of articles were published in the journal Psychoneuroendocrinology (*n* = 99, 11.3%), followed by Stress-the International Journal on the Biology of Stress (*n* = 32, 3.7%), PLOS One (*n* = 28, 3.2%), Physiology & Behavior (*n* = 25, 2.9%), Hormones and Behavior (*n* = 2.5, 2.5%), and Journal of Clinical Endocrinology & Metabolism (*n* = 13, 1.5%) (Table 2). Of the top 10 journals that published the most articles on human salivary cortisol, the top 3 journals with the highest impact factors (IF) were Proceedings of the National Academy of Sciences of the United States of America (IF = 9.4), Neuroscience and Biobehavioral Reviews (IF = 7.5), and Journal of Clinical Endocrinology & Metabolism (IF = 5.0) (Figure 5A). 80% of the 10 journals were categorized as Q2 and above, and 2 as Q3 (Table 2). Among the 10 journals on salivary cortisol with the highest total citations, the most frequently cited journal was PSYCHONEUROENDOCRINO (3,681 citations), followed by J Clin Endocr Metab (1,412 citations). In addition, all of these 10 journals were Q2 or higher except Stree, with Biol Psychiat IF9.6 and P Natl Acad Sci USA IF9.4 (Table 3; Figure 5B).

**Table 2:** The top 10 journals.

Rank	Journal	IF	Q(JCR)	Count	(%)
1	Psychoneuroendocrinology	3.4	2	99	11.3%
2	Stress-The International Journal on the Biology Of Stress	2.6	3	32	3.7%
3	PLOS One	2.9	1	28	3.2%
4	Physiology & Behavior	2.4	2	25	2.9%
5	Hormones and Behavior	2.5	3	22	2.5%
6	Journal of Clinical Endocrinology & Metabolism	5.0	1	13	1.5%
7	Neuroscience and Biobehavioral Reviews	7.5	1	12	1.4%
8	Biological Psychology	2.7	2	11	1.3%
9	Proceedings of the National Academy of Sciences of the United States of America	9.4	1	11	1.3%
10	Scientific Reports	3.8	1	11	1.3%

**Table 3:** The top 10 co-cited journals.

Rank	Journal	Co-citations	IF	Q(JCR)
1	Psychoneuroendocrino	3,681	3.4	2
2	J Clin Endocr Metab	1,412	5.0	1
3	Biol Psychiat	989	9.6	1
4	Psychosom Med	901	2.9	2
5	P Natl Acad Sci USA	617	9.4	1
6	Neurosci Biobehav R	510	7.5	1
7	J Neurosci	496	4.4	1
8	Physiol Behav	492	2.4	2
9	PLOS One	472	2.9	1
10	Stress	462	2.6	3

### 3.4 Authors and co-cited author analysis

**Figure 6:**
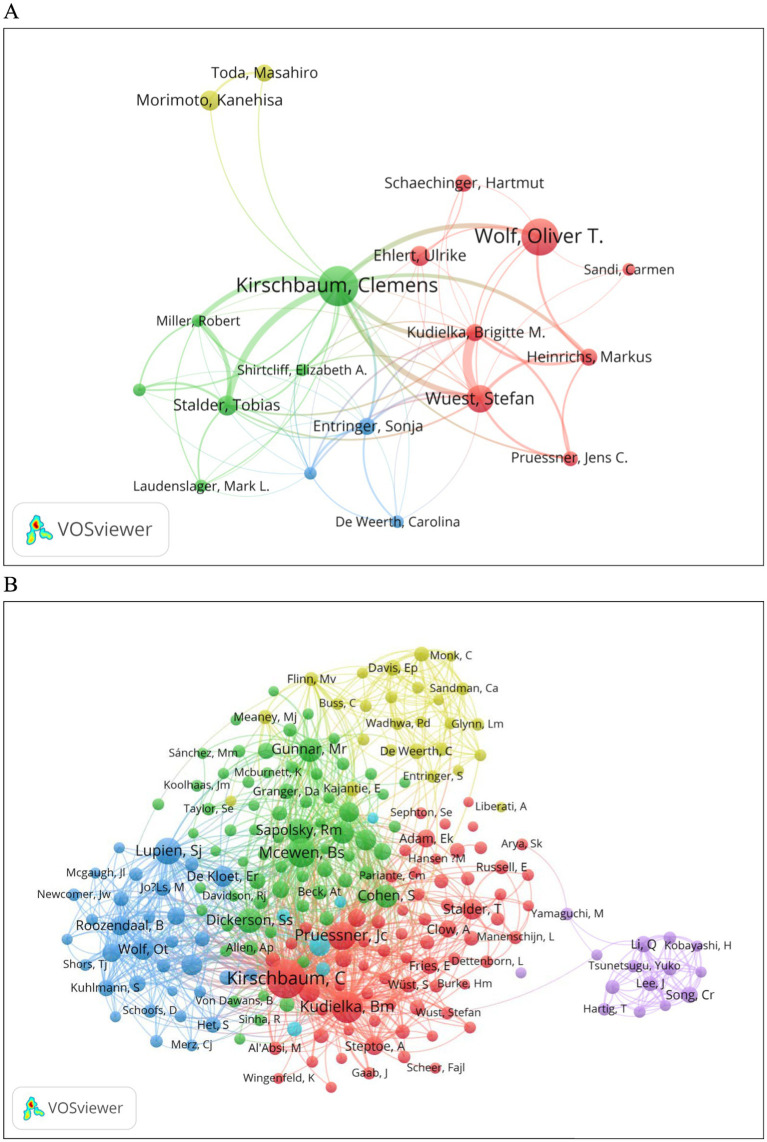
**(A)** Authors. **(B)** Co-cited authors.

From all the authors who have published literature related to human salivary cortisol during the period 2004–2024, the 10 most influential authors are listed in Table 4. The authors who have published the most relevant papers are Kirschbaum, Clemens (*n* = 16), followed by Wolf, Oliver t (*n* = 15). The author collaboration network as shown in Figure 6A shows that Kirschbaum, Clemens, Wolf, Oliver, Wuest, Stefan are located at the key nodes of collaboration for each author, in this neighborhood, in a prominent position. The most prominent nodes are associated with the most published and co-cited authors. The three most co-cited authors are Kirschbaum, C (577), Kudielka, Bm (303) and Mcewen, Bs (265) (Figure 6B; Table 4).

**Table 4:** Ranking of the top 10 authors and co-cited authors.

Rank	Author	Documents	Co-cited author	Citations
1	Kirschbaum, Clemens	16	Kirschbaum, C	577
2	Wolf, Oliver T.	15	Kudielka, BM	303
3	Wuest, Stefan	11	Mcewen, BS	265
4	Ehlert, Ulrike	8	Pruessner, JC	246
5	Morimoto, Kanehisa	8	Lupien, SJ	203
6	Stalder, Tobias	8	Sapolsky, RM	175
7	Entringer, Sonja	7	Dickerson, SS	164
8	Heinrichs, Markus	7	Gunnar, MR	160
9	Kudielka, Brigitte M.	7	Cohen, S	137
10	Schaechinger, Hartmut	7	Yehuda, R	128

### 3.5 Reference and co-cited reference clustering and visual analysis

**Figure 7:**
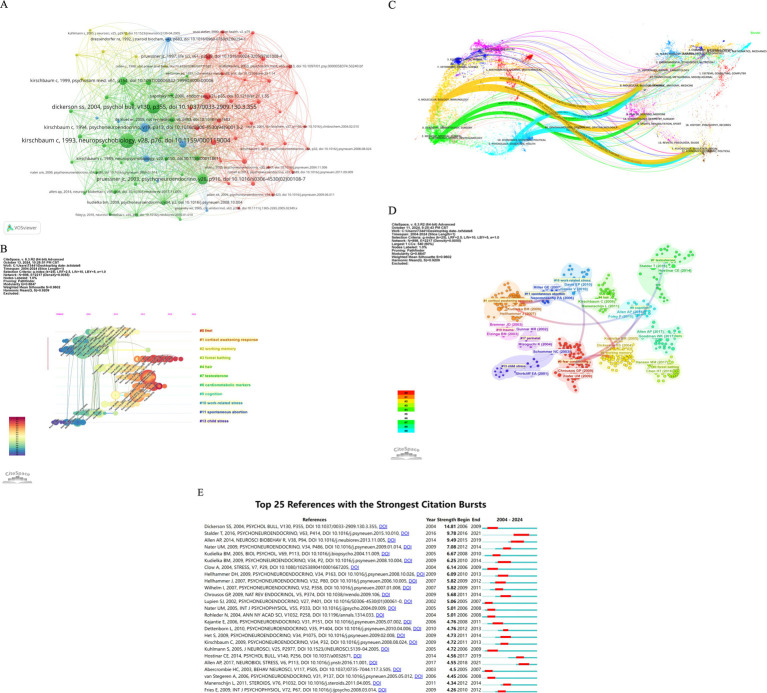
**(A)** Co-cited reference analysis. **(B)** Timeline of cited references. **(C)** Journal double figure overlay. **(D)** Cited references clustering. **(E)** Analysis of citation bursts.

A visualization of the co-cited literature was constructed using the size of the nodes and the strength of the connecting lines (Figure 7A). The ten most frequently cited articles are presented in Table 5, with the article by Kirschbaum, Clemens et al. titled “The ‘Trier Social Stress Test’—a tool for investigating psychobiological stress responses in a laboratory setting” leading with a total of 166 citations, ranking first in both citation count and total link strength. Furthermore, we analyzed the literature in terms of co-citation clusters over time (Figure 7B), resulting in the emergence of 13 distinct clusters. Notably, “child stress” (cluster 13) was identified as the earliest hotspot. Recent years have seen a focus on “testosterone” (cluster 7), “cardiometabolic markers”(cluster 8), and “cognition” (cluster 9) as current hotspots. Additionally, the topics of “cortisol awakening response” (cluster 4) and “working memory” (cluster 2) have garnered significant research attention. Meanwhile, “spontaneous abortion” (cluster 11) has also been extensively studied in recent years, with “forest bathing” (cluster 3) emerging as a new hot topic in this field.

**Table 5:** The cited reference ranked top ten.

Rank	Title	Journal(IF)	Citations	Q(JCR)
1	The ‘Trier Social Stress Test’-a tool for investigating psychobiological stress responses in a laboratory setting	Neuropsychobiology(2.3)	166	1
2	Acute stressors and cortisol responses: a theoretical integration and synthesis of laboratory research	Psychol Bull(17.3)	150	1
3	Two formulas for computation of the area under the curve represent measures of total hormone concentration versus time-dependent change	Psychoneuroendocrino(3.4)	111	2
4	Salivary cortisol in psychoneuroendocrine research: recent developments and applications	Psychoneuroendocrino(3.4)	92	2
5	Impact of gender, menstrual cycle phase, and oral contraceptives on the activity of the hypothalamus-pituitary–adrenal axis	Psychosom Med(2.9)	80	2
6	Salivary cortisol as a biomarker in stress research	Psychoneuroendocrino(3.4)	63	2
7	How Do Glucocorticoids Influence Stress Responses? Integrating Permissive, Suppressive, Stimulatory, and Preparative Actions	Endocr Rev(22)	61	1
8	Salivary cortisol in psychobiological research: an overview	Neuropsychobiology(2.3)	58	1
9	Free cortisol levels after awakening: a reliable biological marker for the assessment of adrenocortical activity	Life Sci(5.2)	55	1
10	A Global Measure of Perceived Stress	J Health Soc Behav(6.3)	51	1

Periodical double graph superposition describes the distribution and direction of topic selection of academic journals. This curve is the cited curve and represents the source of the topic. “Dsycholigy, Education, HEALTH” is derived from “Molecular, Biology, Genetics”, “Psychology, Education, Social.” “MOLECUlar Biology, Immunology” is derived from “Molecular Biology, Genetics,” “Health, Nusing, Medicine”, “Dermatology, Dentistry, Surgery” and “Psychology, Education, Social.” “Molecular, Medical, Clinical” is derived from “Molecular, Biology, Genetics.” “Neurology, Sports, Ophthalmology” is derived from “Dermatology, Dentistry, Surgery” (Figure 7C).

Figure 7D illustrates the clustering of cited literature using Citespace. The connecting lines between the clusters indicate that the red line segment represents the origins of the clusters as early hotspots, while the clusters at the end of the blue line emerged from the earlier clusters at the end of the red line, representing new hotspots. The most cited work is Dickerson and Kemeny (2004) in Psychol Bull, V130, P355, which appears in Cluster #2 with a citation count of 31. The second most cited work is Stalder (2016) in Psychoneuroendocrino, V63, P414, located in Cluster #7, with 23 citations. The third is Allen (2014) in Neurosci Biobehav R, V38, P94, found in Cluster #9 with 19 citations. The fourth is Kudielka (2005) in BIOL PSYCHOL, V69, P113, also in Cluster #2, with 16 citations. The fifth is Kudielka et al. (2009) in Psychoneuroendocrino, V34, P2, located in Cluster #1, with 15 citations. The sixth is Hellhammer (2007) in Psychoneuroendocrino, V32, P80, found in Cluster #1 with 13 citations. The seventh is Hellhammer (2009) in Psychoneuroendocrino, V34, P163, cited 13 times in Cluster #1. The eighth is Clow (2004) in Stress, V7, P29, also cited 13 times in Cluster #1. The ninth is Nater (2009) in Psychoneuroendocrino, V34, P486, found in Cluster #0 with 13 citations. Lastly, the tenth is Chrousos (2009) in Nat Rev Endocrinol, V5, P374, located in Cluster #0 with 12 citations.

### 3.6 Keywords clustering and visual analysis

**Figure 8:**
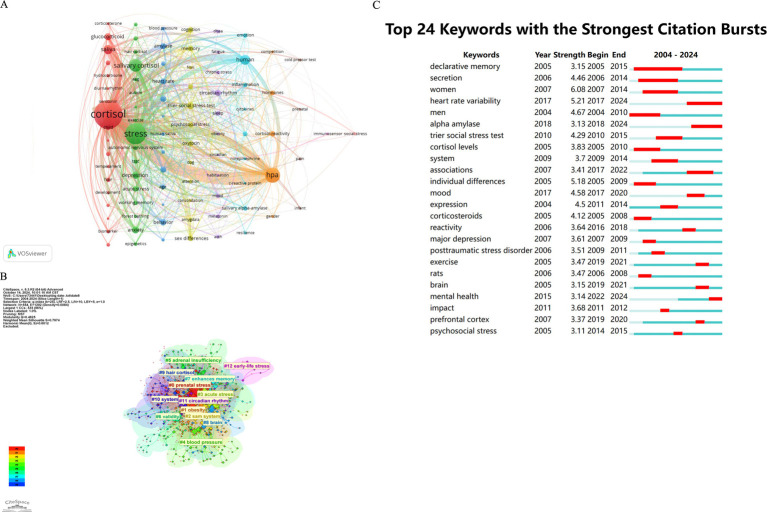
**(A)** Keyword analysis. **(B)** Keyword clustering. **(C)** Concentrated burst of keywords.

We analyzed the frequency of keywords and link strength using VOS viewer software. When the minimum number of occurrences of a keyword was 2, there was a total of 424 keywords. Then we set the threshold to 5 and there were 91 keyword occurrences. The top ten keywords in terms of frequency were cortisol (352), followed by stress (196), HPA (110), salivary cortisol (81), human (50), saliva (44), depression (28), glucocoticoid (28), anxiety (27), and trier social stress test (26) (Figure 8A).

Using CiteSpace fSor keyword clustering, the most cited term in cluster #0 is ‘salivary cortisol,’ which has garnered 444 citations. The second most cited term in the same cluster is ‘HPA,‘with 199 citations. In cluster #1, ‘stress’ ranks third with 174 citations. The fourth position is held by ‘response’ in cluster #2, which has 134 citations. ‘Human’ occupies the fifth position in cluster #3 with 106 citations, while ‘psychosocial stress’ appears twice: first in cluster #3 with 84 citations and again in cluster #2 with 74 citations. ‘Psychological stress’ is noted in cluster #3 with 106 citations and again with 74 citations in the same cluster. Additionally, ‘cortisol’ appears in cluster #8 with 68 citations, followed by ‘gender differences’ in cluster #3 with 59 citations, and ‘depression’ in cluster #1 with 57 citations (Figure 8B).

To identify research frontiers in the field, we employed CiteSpace to analyze burst keywords. Among the top 24 keywords exhibiting the strongest citation bursts, we concentrated on those that began to gain prominence in 2016 (Figure 8C). Notable keywords include “heart rate variability” (burst intensity of 5.21), “reactivity” (burst intensity of 3.64), “exercise” (burst intensity of 3.47), “prefrontal cortex” (burst intensity of 3.37), “brain”(burst intensity of 3.15), “mental health” (burst intensity of 3.14), and “alpha amylase” (burst intensity of 3.13).

**Figure 9:**
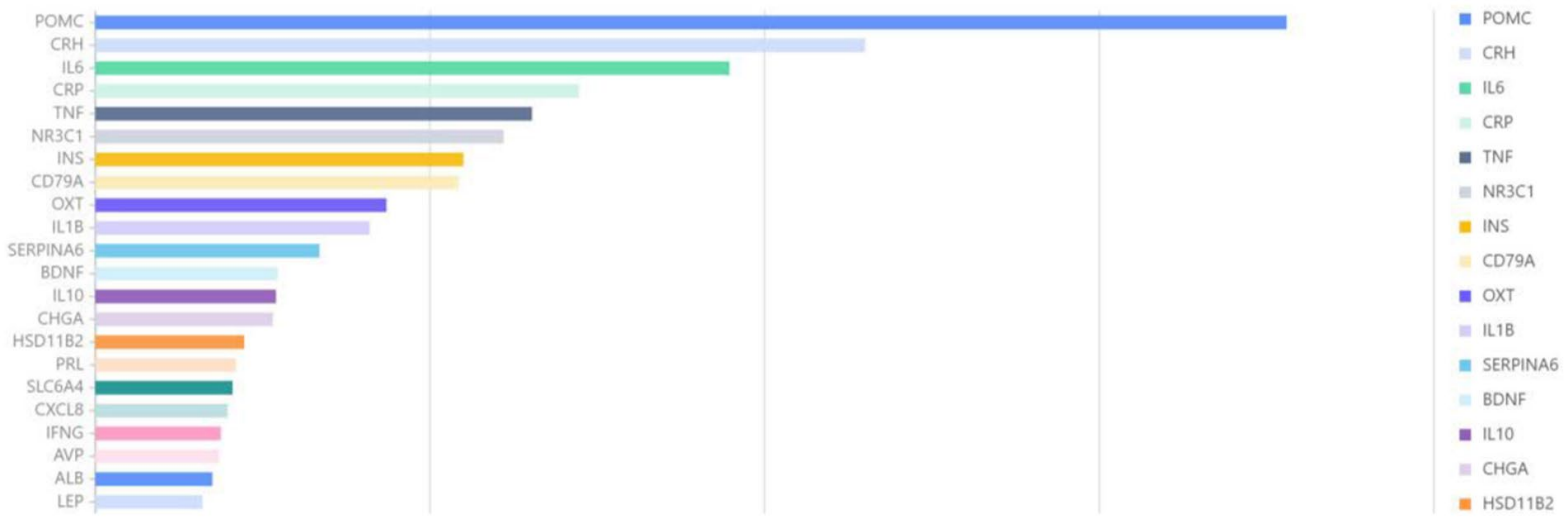
Association gene analysis.

The entity words related to genes in the abstracts of 8,922 articles were extracted and statistically analyzed using the BioBERT biomedical language representation model. As illustrated in Figure 9, POMC was the most frequently documented gene, appearing in 712 articles, followed by CRH with 460 articles, and IL6 with 379 articles.

## 4 Discussion

We conducted a comprehensive search of 876 English articles and review articles concerning human salivary cortisol published in the Web of Science (WOS) from January 1, 2004, to September 30, 2024. Utilizing the Scientific Core Database (SCIE) for analysis, we observed a gradual increase in both the number of publications and citations over this period, indicating a growing interest in this field. The United States leads in the total number of publications and the annual publication rate, underscoring its dominance in this area of research. The top-ranked institution is the University of California System, with 40 publications, highlighting its central role and extensive collaboration with various institutions. The leading countries and institutions in terms of publication volume are predominantly located in developed regions such as North America and Europe, attributed to their early engagement in research, advanced scientific instrumentation, robust economies, and strong international collaboration. China ranks eighth, benefiting from a large population, being the largest developing country, and advancing technological capabilities, suggesting a potential surge in research output in this field in the near future.

The author with the highest number of publications on human salivary cortisol is Clemens Kirschbaum (*n* = 16), followed closely by Oliver Wolf (*n* = 15). The top two co-cited authors are also Kirschbaum (577 citations) and Bm Kudielka (303 citations), indicating that Kirschbaum is the most influential researcher in this field. A total of 1,876 articles have been published across 372 journals, with the highest number of articles appearing in “Psychoneuroendocrinology” (*n* = 99, 11.3%), followed by “Stress: The International Journal on the Biology of Stress” (*n* = 32, 3.7%) and “PLOS One” (*n* = 28, 3.2%). These three journals together account for 18.2% of the total articles published, all of which have Journal Citation Reports (JCR) partitions above zone 3. Among the top ten journals in terms of total citations, “Psychoneuroendocrinology”leads with 3,681 citations, followed by “The Journal of Clinical Endocrinology & Metabolism”(1,412 citations). Notably, “Psychoneuroendocrinology” is the premier journal for both the number of articles published on human salivary cortisol and the total number of citations. To gain a deeper understanding of the research frontiers in this field and to identify potential co-authors, it is advisable to extensively read the articles published in this journal.

Based on the timeline of the cited literature (Figure 6B), which has been categorized into 13 clustered hotspots, we found that early studies primarily focused on “child stress,” “awakening cortisol response,” “working memory, “and “spontaneous abortion.”Mid-term studies included topics such as “fMRI,” “spontaneous abortion, “and “working memory,” while the current focus has shifted towards “testosterone,” “cardiometabolic markers,” and “forest bathing”. Additionally, we conducted an analysis of the top ten most cited articles. An examination of their titles revealed that “stress” was frequently mentioned, and salivary cortisol is recognized as a biomarker of stress (Sharma et al., 2024). This indicates that salivary cortisol, as the end product of the HPA axis, is closely related to human psychological states and regulates endocrine and immune functions (Pulopulos et al., 2020). The relationship between salivary cortisol and psychological states, along with its association with disease development, has been a prominent research topic for decades. Furthermore, we analyzed the outbreak intensity of the top 25 articles in the cited literature (Figure 7E). Notably, Sally S. Dickerson published “Acute Stressors and Cortisol Responses: A Theoretical Integration and Synthesis of Laboratory Research,” which had an outbreak intensity of 14.81 and ranked first, demonstrating its significant influence and recognition among scholars in the field. This article focuses on a meta-analysis of 208 laboratory studies on acute psychological stressors and tests a theoretical model that describes the conditions triggering a cortisol response, concluding that psychological stressors increase cortisol levels. The magnitude of the cortisol effect is contingent upon the characteristics of the stressor; performance tasks that involve social appraisal of threat and/or uncontrollability result in significant elevations in cortisol levels (Dickerson and Kemeny, 2004). Stress is recognized as a prominent risk factor for adverse health outcomes, with increased salivary cortisol concentrations serving as a physiological response to acute psychosocial stress (Fan et al., 2024). Salivary cortisol is also regarded as a non-invasive biomarker of stress (Vasanthi et al., 2022). Numerous scholars have developed methodologies to assess variations in human stress levels through the measurement of salivary cortisol, encompassing diverse populations such as athletes (Tsunekawa et al., 2023), workers (Vanttola et al., 2024), children (Lai et al., 2020), teachers (Pondeljak et al., 2023), and patients (Rahate et al., 2022; Guarnotta et al., 2024; Teo et al., 2023). Fluctuations in salivary cortisol levels are indicative of emotional states, aid in disease progression detection, and serve as crucial monitoring molecules.

To investigate the academic hotspots and topics related to human salivary cortisol, we employed Citespace software to analyze the keyword outbursts (Figure 8C), which revealed a total of 24 significant keywords. In the early phase, the prominent keywords included “men,” “women,” “declarative memory,” “individual differences,” “secretion,” “cortisol levels,” “corticosteroids,” “rats”, and “major depression.” In the middle phase, the key terms were “trier social stress test,” “prefrontal cortex,” “psychosocial stress,” “exercise,” “system,” “dominance,” “impact,” “mood,” “associations,” and “posttraumatic stress disorder.” Currently, the focal areas include “mental health,” “alpha amylase,” and “heart rate variability”.

Analyzing the pre-explosive words, we found that early hotspots concentrated on the relationship between factors such as gender (Lederbogen et al., 2010), work stress (Špiljak et al., 2022), depression (Doan et al., 2024), declarative memory (Maheu et al., 2005), individual differences (Sgoifo et al., 2003), and salivary cortisol secretion. The results confirmed that these factors significantly influence the level of salivary cortisol. The relationship between corticosteroid hormones (cortisol in humans and corticosterone in rodents) and the role of memory modulation during emotional arousal and stressful experiences has been investigated. When these hormones are activated during emotional arousal and stressful experiences, they affect declarative memory through their interactions with corticosteroid receptors located in the frontal lobe, amygdala, and hippocampus. Declarative memory is related to HPA axis function and cardiovascular regulation. Some foreign institutions have predicted a negative correlation between declarative memory performance and salivary cortisol response, indicating that increased salivary cortisol may be accompanied by diminished declarative memory (Wright et al., 2005). Early scholars conducted studies on corticosterone in rodents, such as mice, for scientific experiments since primates like humans release cortisol while rodents release corticosterone. Both molecules are potent glucocorticoids and are considered physiological markers of mammalian HPA axis activity, exhibiting similar effects (Selye, 1973). Cushing’s syndrome is secondary to pro-adrenocortisolism, and early researchers identified salivary cortisol levels at 11 p.m. as a modern and simple initial screening tool for diagnosing Cushing’s syndrome. However, in patients with recurrent ambiguous results, they discovered that reassessment after several months or corticotropin-releasing hormone (CRH) stimulation testing following low-dose dexamethasone suppression could help rule out a pseudo-Cushing state (Gross et al., 2007).

With the continuous development of the economy and society, as well as advancements in science and technology, scholars have conducted extensive research on salivary cortisol. The relationship between the prefrontal cortex and salivary cortisol has emerged as a prominent research topic, particularly concerning the correlation between the structure of the medial prefrontal cortex and cortisol levels in elderly men and women (Stomby et al., 2016). Increased psychosocial stress predisposes individuals to anxiety and is accompanied by changes in salivary cortisol concentrations. A moderate positive correlation has been identified between salivary cortisol and anxiety responses to social stress, as evidenced by the Trier Social Stress Test (Grace et al., 2022). Moreover, exercise has been shown to reduce cortisol concentrations during psychosocial stress (Wood et al., 2018). Mood alterations, such as anxiety and depression, are associated with dysfunction of the HPA axis and emotional processing endpoints. Cortisol, the primary physiological end product of the HPA axis, is typically linked to stress and negative affect. Research indicates that HPA axis activity may selectively interfere with the support of emotional perception, processing, and regulation, with salivary cortisol activity being associated with neural processing of emotional information (Peters et al., 2016). Growing evidence suggests that salivary cortisol plays an active role in regulating emotions in daily life (Hoyt et al., 2016). Furthermore, individuals who have experienced traumatic stress disorder often exhibit altered salivary cortisol concentrations, with many studies indicating that patients with post-traumatic stress disorder (PTSD) demonstrate lower fluctuations in cortisol levels (Wahbeh and Oken, 2013). Some researchers have reported lower salivary cortisol levels in females with dominant samples compared to those with non-dominant samples (Gonzalez-Santoyo et al., 2015). Additionally, family socioeconomic status uniquely influences sleep and HPA axis functioning in adolescents, with salivary cortisol levels showing a strong correlation with family economic and social status (Rocha et al., 2022). Notably, low salivary cortisol levels during preadolescence, when adolescents are at rest, are associated with more aggressive behavior in late adolescence (Shoal et al., 2003). Dutch scholars have discovered that psychological states, such as despair in response to sadness, are consistently linked to higher salivary cortisol arousal responses (van Santen et al., 2011). Foreign researchers have found that work stress and the expression of extrinsic anger correlate with elevated salivary cortisol concentrations during the early workday (Steptoe et al., 2000). The presence of gender differences in both early and mid-term studies highlights the complexity of this field; however, variations in experimental purpose, research methodology, and comparison objectives have led to differing results. In conclusion, the comprehensive examination of the research on salivary cortisol, encompassing socioeconomic status, gender differences, human aggression, dominance, psychological state, and emotional expression, illustrates that salivary cortisol, as a signaling molecule, exhibits a close correlation across diverse research contexts.

Current research focuses on the interrelationship between mental health, *α*-amylase, and heart rate variability. This study investigates the connections between the HPA axis and the autonomic nervous system (ANS), particularly the physiological responses of these systems to perceived threats. Salivary cortisol is recognized as a peripheral marker of HPA axis activity (Schwartz et al., 1998), while salivary α-amylase (sAA) has been established as a reliable peripheral marker of ANS activity (Granger et al., 2007). Experimental studies have demonstrated that salivary α-amylase and salivary cortisol interact in response to perceived threats, highlighting their roles within the ANS and HPA axis (Granger et al., 2007; Nater and Rohleder, 2009; Ursache and Blair, 2015). Scholars now regard salivary cortisol, as the end product of the HPA axis, as a crucial marker for assessing a patient’s mental health status (Kunasegaran et al., 2023; Kolbe et al., 2023; Kaligis et al., 2023; Dagli et al., 2024; Ilen et al., 2024). Salivary cortisol exhibits physiological rhythms and circadian variations, which can be influenced by extrinsic factors, leading to abnormal fluctuations in different populations. Measuring salivary cortisol concentrations at various times throughout the day and night is an effective approach to determine potential risks to a patient’s mental health. Monitoring salivary cortisol levels at different times may be vital for disease diagnosis, treatment, prevention, and the development of innovative therapeutic methods (Wang et al., 2023; Berdina et al., 2022; Nishimi et al., 2022; Tsunekawa et al., 2022; Ballerini et al., 2024). Salivary cortisol serves not only as an important biomarker but also as a biological stress molecule, facilitating evaluations of stress across various diseases and environmental contexts (Hirokawa et al., 2022), including anxiety and depression (McLaughlin et al., 2022). Furthermore, these assessments can be integrated with heart rate variability measurements to evaluate changes in stress levels (Mathobela et al., 2024). For instance, they can help identify psychological disturbances in patients with burning mouth syndrome and temporomandibular joint disorder (He et al., 2024; Klepzig et al., 2024), thereby providing valuable insights for interdisciplinary treatment. Additionally, salivary cortisol testing can assist in diagnosing adrenocortical insufficiency and Cushing’s syndrome (Kvam Hellan et al., 2024; Efthymiadis et al., 2024), laying a theoretical foundation for advancements in testing instruments and technologies.

Salivary cortisol is widely utilized in clinical research for stress assessment, endocrine disease diagnosis, and psychiatric disease studies. However, its application is accompanied by numerous controversies and limitations. These include the standardization of detection methods, as the reference intervals for ELISA may differ from those established by chemiluminescence methods. Additionally, significant variations in sampling time points can limit the comparability of results. The stability of results may also be influenced by different storage conditions, such as freezing and centrifugation. For instance, cortisol secretion is affected by multiple factors, including circadian rhythm, stressful events, sleep quality, and interpersonal relationships (Giannouli, 2017). Insomnia or sleep deprivation can elevate salivary cortisol levels, complicating the diagnosis of depression or anxiety. If the timing or environment is not rigorously controlled during research design (Taylor, 2012), confounding factors may be introduced. This variability is evident in the contradictory findings regarding salivary cortisol in depression studies; while several studies support a correlation between salivary cortisol and depression, the results remain heterogeneous. Meta-analyses indicate that salivary cortisol levels in patients with depression are significantly higher than those in healthy controls, particularly upon waking, suggesting a positive association (Miller and Kirschbaum, 2019). Conversely, some research has reported no significant changes in salivary cortisol levels following antidepressant treatment, raising questions about its reliability as a marker of treatment efficacy. Furthermore, certain subtypes of depression, such as atypical depression, may be associated with decreased cortisol levels.

Salivary cortisol has demonstrated significant potential for clinical applications, yet it also presents certain limitations. Various studies have proposed differing diagnostic thresholds, leading to inconsistencies in their application. Regarding disease specificity, the performance of salivary cortisol is inadequate; its elevation is not exclusively indicative of depression and anxiety, as it may also occur in metabolic disorders such as obesity and diabetes. Therefore, methodological standardization, individual variability, and external factors must be meticulously considered in its application. Looking ahead, multi-center collaboration, technological innovation, and interdisciplinary integration will be essential to enhance its reliability and clinical utility.

One of the strengths of this study is the use of the wos database, which is widely recognized for its comprehensive coverage of the medical literature and its high quality bibliographic content, as well as its multiple analytical tools. By utilizing wos, this analysis ensured a solid foundation of scholarly articles focusing on the study of human salivary cortisol, enhancing the reliability and breadth of the bibliometric findings. The study utilized a combination of qualitative and quantitative methods, such as a thematic analysis of keyword co-occurrence and literature co-occurrence. This dual approach quantified publication trends and author collaboration, and identified and explained thematic clusters in the literature. By combining qualitative insights with quantitative metrics, the study provided a nuanced understanding of human salivary cortisol research themes and directions. The rigor of this approach enhances the comprehensiveness and depth of the bibliometric analysis, providing valuable insights into scholarly discourse and informing future research efforts. To our knowledge, no other bibliometric analysis has been done on this topic. While this bibliometric analysis provides valuable insights into the research landscape surrounding human salivary cortisol, some limitations must be recognized. First, this study utilized only data from the wos database, and a single database may not accurately and comprehensively reflect the research in this area. In addition, the survey was largely quantitative, focusing on metrics such as publication frequency and authorship patterns without assessing the quality of individual papers, making it difficult to obtain deeper research. Despite these limitations, the study provides a comprehensive overview of the research landscape in the field, offering valuable guidance to emerging researchers and enhancing understanding of the subject matter.

### 4.1 Recommendations for future research

Future research on salivary cortisol in humans should prioritize larger and more extensive clinical trials. The influence of lifestyle factors and comparative studies across cultures and population diversity are crucial. Furthermore, advanced analytical techniques, real-time monitoring technologies, standardized methods, and quality assessments of individual studies will enhance the reliability and comprehensiveness of the results. The relationship between salivary cortisol and psychosomatic disorders, oral diseases, cardiovascular system diseases, and endocrine system disorders has been a research hotspot. However, many unexplored areas remain, such as utilizing the circadian rhythmicity of salivary cortisol to develop advanced detection instruments and applying cognitive behavioral therapy to monitor salivary cortisol levels. Additionally, developing personalized mental health care solutions is essential. In modern society, mental health is increasingly important, as depression, anxiety, and other psychological factors significantly contribute to various diseases. This necessitates collaborative efforts between researchers and institutions to implement psychotherapy and adopt a multidisciplinary approach to treatment. Promoting interdisciplinary research and innovation will aid in exploring the role of human salivary cortisol in disease discovery, prevention, and treatment, ultimately leading to new breakthroughs that improve treatment outcomes and enhance quality of life.

## 5 Conclusion

This review summarizes 876 articles on human salivary cortisol published between 2004 and 2024, providing insights into the current state of the field and predicting future research directions. It is the first article to employ bibliometric methods to summarize human salivary cortisol research. The bibliometric analysis reveals extensive collaboration and participation globally, encompassing a total of 60 countries, 372 journals, and 4,317 authors. The United States leads in both the annual and total number of articles published, underscoring its dominance in this field. The journal “Psychoneuroendocrinology” published the highest number of articles, totaling 99. The author with the most publications in this domain is Clemens Kirschbaum, with 16 articles. In the keyword clustering analysis, cortisol received the highest number of citations, totaling 352. The most cited article, “The ‘Trier Social Stress Test’ – a tool for investigating psychobiological stress responses in a laboratory setting, “garnered 166 citations. In conclusion, salivary cortisol is a significant biomarker and a crucial stress molecule. Its role warrants ongoing exploration across various domains, including the investigation of molecular mechanisms, disease diagnosis, the development of research instruments, longitudinal clinical studies, interdisciplinary linkages, and other emerging areas that represent hotspots for future research.
